# The Menin Tumor Suppressor Protein Is Phosphorylated in Response to DNA Damage

**DOI:** 10.1371/journal.pone.0016119

**Published:** 2011-01-14

**Authors:** Joshua Francis, Wenchu Lin, Orit Rozenblatt-Rosen, Matthew Meyerson

**Affiliations:** 1 Department of Medical Oncology, Dana-Farber Cancer Institute, Harvard Medical School, Boston, Massachusetts, United States of America; 2 Department of Pathology, Harvard Medical School, Boston, Massachusetts, United States of America; University Medical Center Hamburg-Eppendorf, Germany

## Abstract

**Background:**

Multiple endocrine neoplasia type 1 (MEN1) is a heritable cancer syndrome characterized by tumors of the pituitary, pancreas and parathyroid. Menin, the product of the *MEN1* gene, is a tumor suppressor protein that functions in part through the regulation of transcription mediated by interactions with chromatin modifying enzymes.

**Principal Findings:**

Here we show menin association with the 5′ regions of DNA damage response genes increases after DNA damage and is correlated with RNA polymerase II association but not with changes in histone methylation. Furthermore, we were able to detect significant levels of menin at the 3′ regions of *CDKN1A* and *GADD45A* under conditions of enhanced transcription following DNA damage. We also demonstrate that menin is specifically phosphorylated at Ser394 in response to several forms of DNA damage, Ser487 is dynamically phosphorylated and Ser543 is constitutively phosphorylated. Phosphorylation at these sites however does not influence the ability to interact with histone methyltransferase activity. In contrast, the interaction between menin and RNA polymerase II is influenced by phosphorylation, whereby a phospho-deficient mutant had a higher affinity for the elongating form of RNA polymerase compared to wild type. Additionally, a subset of MEN1-associated missense point mutants, fail to undergo DNA damage dependent phosphorylation.

**Conclusion:**

Together, our findings suggest that the menin tumor suppressor protein undergoes DNA damage induced phosphorylation and participates in the DNA damage transcriptional response.

## Introduction

Menin, the product of the multiple endocrine neoplasia type 1 (*MEN1*) gene is a tumor suppressor protein whose mechanism of action is not completely understood [1], [2]. Germline mutations of *MEN1* result in an autosomal dominant syndrome characterized by tumors of the endocrine pancreas, the anterior pituitary, and the parathyroid glands [3]. Somatic mutations of the *MEN1* gene have also been described in neuroendocrine tumors and arise when the second wild-type allele has undergone a loss of heterozygosity [4], [5], [6]. While *MEN1* mutations are only tumorigenic within neuroendocrine lineages, menin is expressed in most tissues, at all stages of development and likely has a universal function [7], [8]. Sequence analysis of menin protein reveals a high degree of conservation amongst metazoans; however, no obvious functional domains have yet been identified [9].

Menin has been reported to interact with a broad spectrum of proteins, most of which participate in the regulation of transcription (see reviews for details [10], [11]. An important insight into understanding the tumor suppressor functions of menin occurred when it was found that menin interacts with a SET1-like histone methyltransferase complex containing KMT2A (MLL) and KMT2D (MLL4 originally known as MLL2) [12], [13]. Men1^+/-^ mice that have undergone loss of the wild-type allele within the pancreatic islet develop islet cell hyperplasia and insulinomas, a phenotype that mirrors the human disease [14]. In these islets, there is a failure to recruit the histone methyltransferase complex to the cyclin-dependent kinase inhibitor genes *CDKN2C* (p18) and *CDKN1B* (p27) leading to an attenuation of transcription and an increase in proliferation [14]. Surprisingly, menin is also required in the formation for MLL-fusion induced leukemias, where it is thus acting in a pro-oncogenic manner [15].

Genome-wide chromatin studies have found menin to be associated with promoter and coding regions of hundreds of genes suggesting a more general function in transcription [16], [17]. While these results are compelling, studies by several laboratories have shown that a portion of MEN1-associated missense point mutants retain the ability to interact with KMT2A/KMT2D suggesting an additional function or functions for menin tumor suppressor activity outside of histone methylation [12], [18]. Recently, menin was found to function with SKIP and c-Myc in HIV-1 Tat-mediated transcription which was independent of KMT2A (MLL) [19].

The DNA damage response is an intricate cellular system that coordinates the repair of DNA damage, the activation of cell-cycle checkpoints to facilitate repair, and apoptosis in order to eliminate cells with extensive DNA damage [20]. Menin has often been implicated in the DNA damage response and studies using Men1^-/-^MEFs have shown that these cells are hypersensitive to intrastrand crosslinking agents and fail to activate cellular checkpoints after γ-irradiation (γ-IR) [21], [22]. Menin has been found to interact with p53-containing complexes and to function as a transcriptional activator of *CDKN1A* (p21) after γ-IR treatment [22], [23]. Several pieces of evidence also support a role for menin in DNA repair including interactions with FANCD2 and RPA [21], [24]. Men1 deficient Drosophila mutants were found to be hypermutable in response to DNA damage [25]. Menin is phosphorylated at two Ser residues, Ser543 and Ser583, and it is possible that post-translational modifications may mediate distinct cellular functions [26].

In this study we describe menin association with 5′ regions of DNA repair and cell cycle genes in the absence of DNA damage. Following DNA damage, we observed a significant increase in menin association with these genes at both the 5′ and 3′ regions. We also confirm that menin is phosphorylated in response to DNA damage at Ser394 and describe a novel phosphorylation site at Ser487 that is dynamically regulated following different forms of DNA damage. A subset of MEN1-associated missense point mutants failed to become phosphorylated after DNA damage and may link altered tumor suppressor function to the DNA damage response. The post-translational modification of menin may play an as yet undetermined functional role in the tumor suppressor function of menin.

## Results

### Menin is associated with the 5′ regions of genes involved in DNA repair

**Figure 1:**
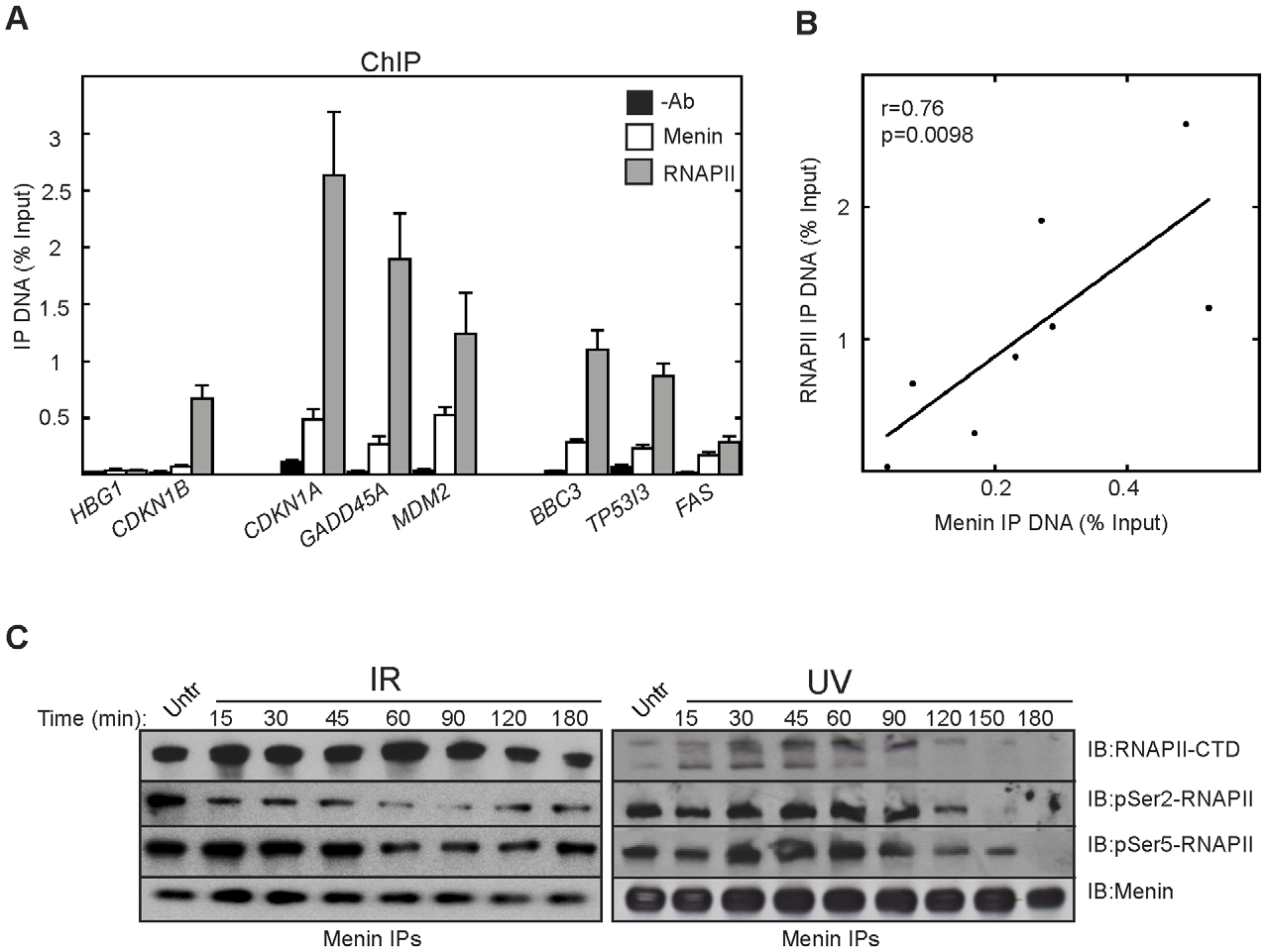
Menin is associated with the 5′ regions of DNA damage responsive genes in the absence of damage. (A) ChIPs were performed in untreated U2OS cells with antibodies for menin, or RNA polymerase II (RNAPII) or no antibody (-Ab) as a background control. Precipitated chromatin was used for quantitative real-time PCR using primers that amplify 5′ regions of the indicated genes. Results are the average of at least 3 independent experiments and represent the amount of DNA immunoprecipitated with each antibody relative to the amount of input DNA. Error bars indicate standard error of the mean. (B) Scatter plot showing the correlation between menin and RNAPII at the 5′ regions of the indicated genes. (C) Menin immunoprecipitations (IPs) were performed with anti-menin antibody over the course of 3 hours in 293T cells treated with either 1000 Rads of γ-IR or 25J/m^2^ UV. The resulting immunocomplexes were resolved and immunoblotted with antibodies targeting phosphorylated residues within the carboxyl-terminal domain (CTD) of RNAPII.

The tumor suppressor protein menin has been best characterized as a transcription regulatory factor. We utilized chromatin immunoprecipitation (ChIP) assays as a means to investigate a role for menin in the DNA damage response. The DNA damage induced transcription response is mediated in large part through the tumor suppressor p53. Upon activation and stabilization, p53 can increase the expression of genes involved in cell-cycle arrest, DNA repair and apoptosis among others [27]. ChIP assays in the p53 wildtype expressing cell line U2OS revealed that even in the absence of DNA damage, menin is found to associate with the 5′ regions of genes involved in cell cycle control (*CDKN1A* and *GADD45A*), p53-regulation (*MDM2*) and apoptosis (*BBC3*, *TP53I3*, and *FAS*) (Figure 1A). A very low amount of detectable menin was associated with the previously identified target gene *CDKN1B* suggesting a cell-type specific role for menin association with this locus [14]. The non-expressed hemoglobin *HBG1* gene is shown for comparison. While varying amounts of RNA polymerase II (RNAPII) were observed at the 5′ regions of these genes, a strong correlation exists between menin and RNAPII association at these genes (Figure 1B).

We next sought to examine the interaction between menin and RNAPII before and after DNA damage. Previous co-immunoprecipitation studies had revealed that menin was able to interact with RNAPII that was phosphorylated at Ser5 within the carboxyl-terminal domain (CTD) of the large subunit of RNAPII and more weakly with RNAPII phosphorylated at Ser2 of the CTD [12], however by including a sonication step in the lysate preparation we were now able to see a more robust interaction between menin and RNAPII phosphorylated at Ser2 (Figure 1C). The interaction between menin and RNAPII was present before treatment (untreated) and remained intact for at least 3 hours following γ-irradiation (γ-IR). Interestingly, the interaction between menin and pSer2-RNAPII is reduced within 15 minutes and remains diminished until about 2 hours post-exposure. During the same timecourse after γ-IR, the interaction between menin and pSer5-RNAPII is initially increased followed by a gradual decline. However exposure to 25 J/m^2^ of ultraviolet (UV) irradiation resulted in a transient increase in the association of menin and pSer2- and pSer5-RNAPII until approximately 2 hours, after which time the interaction was diminished presumably due to UV-induced RNAPII degradation (Figure 1C).

### DNA damage triggers an increase in menin association at DNA damage responsive genes

**Figure 2:**
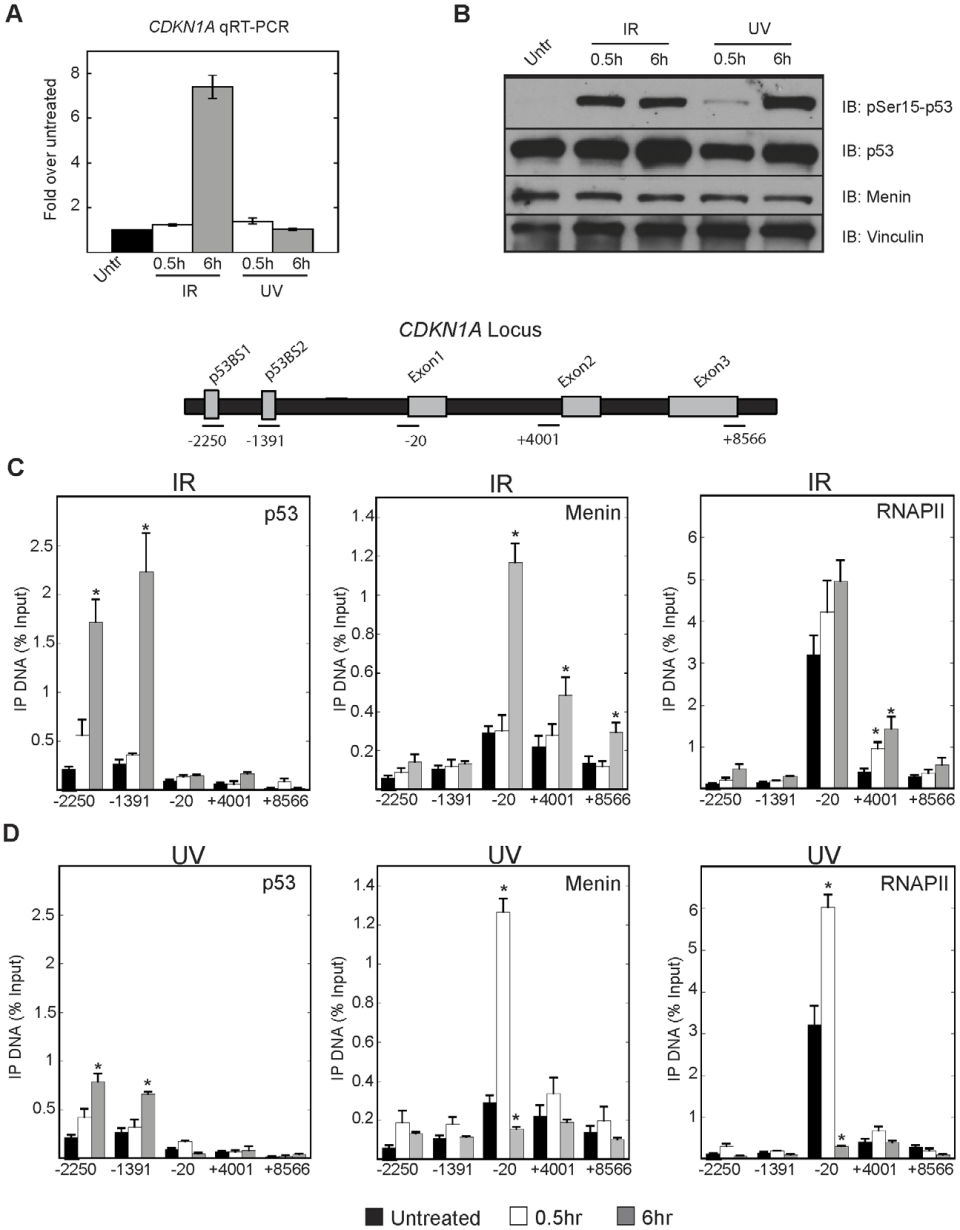
Menin is associated with the *CDKN1A* locus after DNA damage. (A) Quantitative RT-PCR results showing induction of *CDKN1A* mRNA 0.5 h or 6 h following treatment with 1000 Rads of γ-IR or 10J/m^2^ UV in U2OS cells. (B) Whole cell extracts were prepared from U2OS cells under the above conditions and immunoblotted with the indicated antibodies. ChIPs were performed from U2OS cells following treatment with 1000 Rads of γ-IR (C) or 10J/m^2^ UV (D). p53 (left panel), menin (middle panel) and RNAPII (right panel) were quantitated along the *CDKN1A* locus. Results are the average of at least 3independent experiments. Error bars are standard error of the mean. * p<0.05.

After finding a correlation between menin and RNAPII association with DNA damage response genes, we next wanted to look at the effects DNA damage would have on menin localization. RNA was harvested from U2OS cells treated with 1000 Rads of γ-IR or 10 J/m^2^ UV after 0.5 h and 6 h to look at time dependent changes in transcription. Quantitative RT-PCR showed that the mRNA levels of the cell cycle regulator Cyclin-Dependent Kinase Inhibitor 1A gene (*CDKN1A*) were elevated 7-fold at 6 hours after γ-IR treatment while we were unable to detect a change in *CDKN1A* mRNA after UV (Figure 2A). Immunoblots of U2OS cells under the same conditions show phosphorylation of p53 at Ser15 indicating that the DNA damage response was initiated following γ-IR and UV treatment (Figure 2B).

We next performed ChIP after γ-IR and UV treatment to understand differences in early and late menin association with the *CDKN1A* locus. Following γ-IR, a time dependent increase in menin association was observed at the proximal promoter that correlated with increased p53 association at the upstream p53 binding sites (Figure 2C). UV treatment revealed a strikingly different pattern in association whereby an increase in menin was found after a short incubation time and was dramatically reduced after 6 hours mirroring what was observed for RNAPII (Figure 2D). Interestingly, we were able to consistently detect an association of menin along the length of the locus under conditions of high expression. Histone H3 tri-methyl-lysine 4 (histone H3-K4me3) levels 6 hours after γ-IR were not significantly altered (Figure S1).

**Figure 3:**
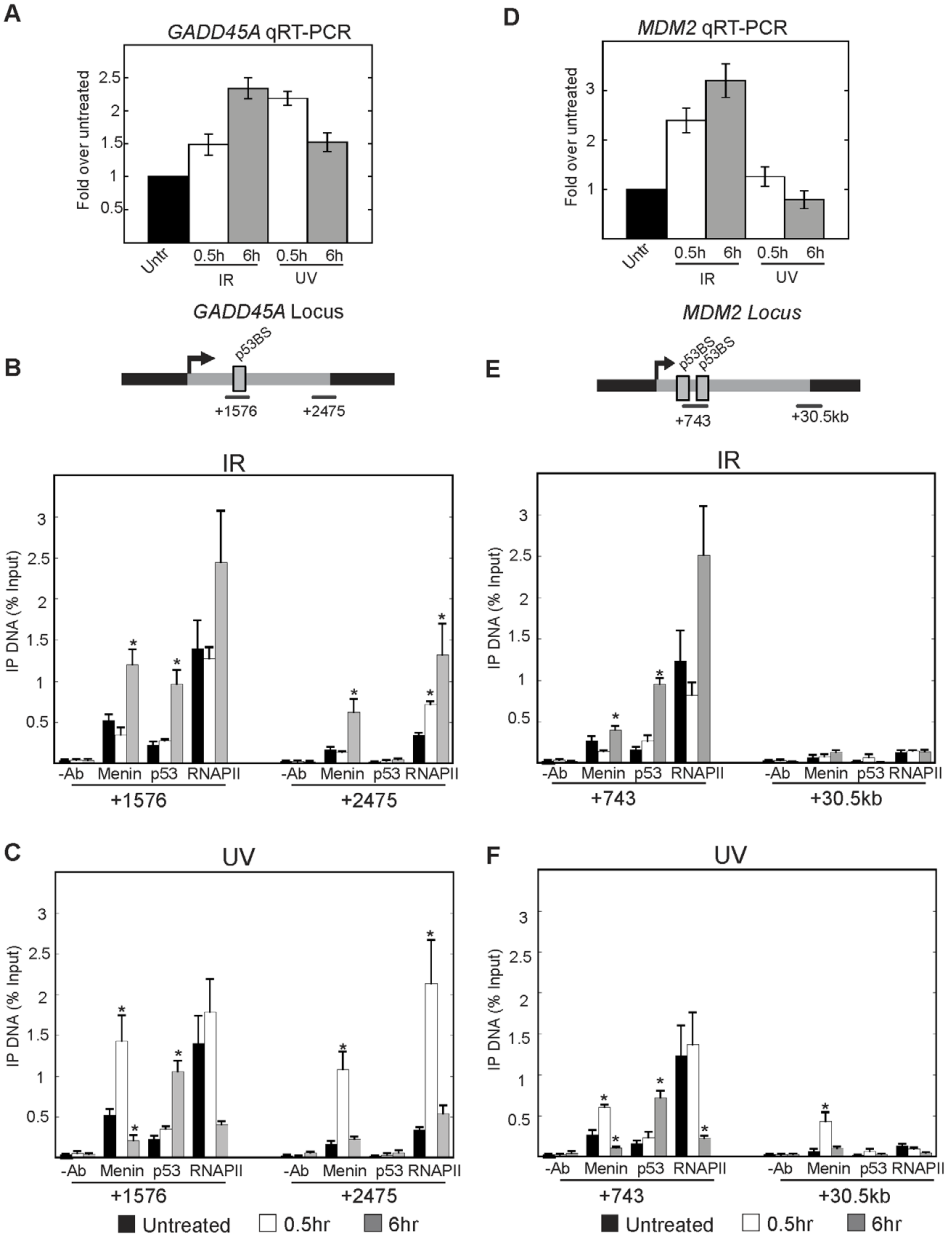
Menin is enriched at additional DNA damage responsive genes after DNA damage. Quantitative RT-PCR results showing induction of *GADD45A* (A) and *MDM2* (D) mRNA 0.5 h or 6h following treatment with 1000 Rads of γ-IR or 10J/m^2^ UV in U2OS cells. ChIPs were performed in U2OS cells under the above conditions. The association of menin, p53 and RNAPII with the *GADD45A* locus was quantitated following treatment with 1000 Rads of γ-IR (B) or 10J/m^2^ UV (C). ChIP of menin, p53, and RNAPII along the *MDM2* locus after IR (E) or UV (F). Graphs represent the amount of DNA immunoprecipitated with each antibody relative to the amount of input DNA. Results are the average of at least 3 independent experiments. Error bars are standard error of the mean. * p<0.05.

To further examine the role of menin in DNA damage-dependent transcription, we looked at menin association along two additional DNA damage responsive genes including, the Growth Arrest and DNA-Damage-inducible Alpha gene (*GADD45A*) and the Mouse Double Minute 2 (*MDM2*) gene. *GADD45A* mRNA levels were found to be quickly elevated following both γ-IR and UV treatment (Figure 3A). ChIP studies revealed that menin was significantly increased at the *GADD45A* locus 6 hours after γ-IR and 30 min after UV, consistent with a gene undergoing induction of expression (Figure 3B and C).

Analysis of *MDM2* expression revealed an increase in mRNA levels after γ-IR and only a modest short-term increase after UV treatment (Figure 3D). ChIP studies found that menin was significantly enriched at the 5′ regions of the *MDM2* locus 6 hours after treatment with γ-IR and 30 min after UV treatment (Figure 3E and F). Menin association with the 3′ region of the *MDM2* locus was observed after UV treatment but not following γ-IR (Figure 3E and F).

We were able to use shRNA to knockdown menin in U2OS cells, however we were unable to consistently see an effect on *CDKN1A*, *MDM2* or *GADD45A* transcription following DNA damage. One possible interpretation of the lack of transcriptional consequences of *MEN1* knockdown may be attributed to the off-target effects of *MEN1* RNAi described previously [28].

### Menin is phosphorylated in response to DNA damage

We next wanted to explore whether post-translational modifications could be mediating the increases in menin association with specific gene loci after DNA damage. Large-scale mass spectrometry studies found menin to be phosphorylated at Ser394 (Ser399 in isoform1) in response to γ-IR and UV treatment [29], [30]. To expand upon this finding, we immunoprecipitated endogenous menin from 293T cells following treatment with different means of inducing DNA damage and then used mass spectrometry to identify phosphorylation sites. DNA damage-specific phosphorylation was detected at Ser394, a residue in the region containing the SQ/TQ cluster. A new phosphorylation site at Ser487 was identified in all samples regardless of DNA damage as well as the previously reported site at Ser543 (Figure S2) [26].

Ser394 resides within an SQ/TQ cluster, the motif recognized by DNA damage transducing kinases. To ascertain whether or not nearby Thr397 or Ser399 were also phosphorylated, we generated the Ser394Ala substitution and performed an immunoprecipitation on Flag-tagged-Ser394Ala menin purified after treatment with the topoisomerase II inhibitor adriamycin (Adr). Mass spectrometric analysis revealed the presence of a phospho-peptide within the cluster suggesting that either Thr397 or Ser399 or both may also be phosphorylated after DNA damage. This finding is important because Ser394 is conserved in larger mammals but not conserved in rodents (Figure S2A and S2C). Mass spectrometry also detected phosphorylation at the conserved Ser487 and Ser543 sites on menin immunopurified from the mouse islet β-cell line βTC3 under normal cell culture conditions suggesting that these sites may be functionally important (Figure S2B).

**Figure 4:**
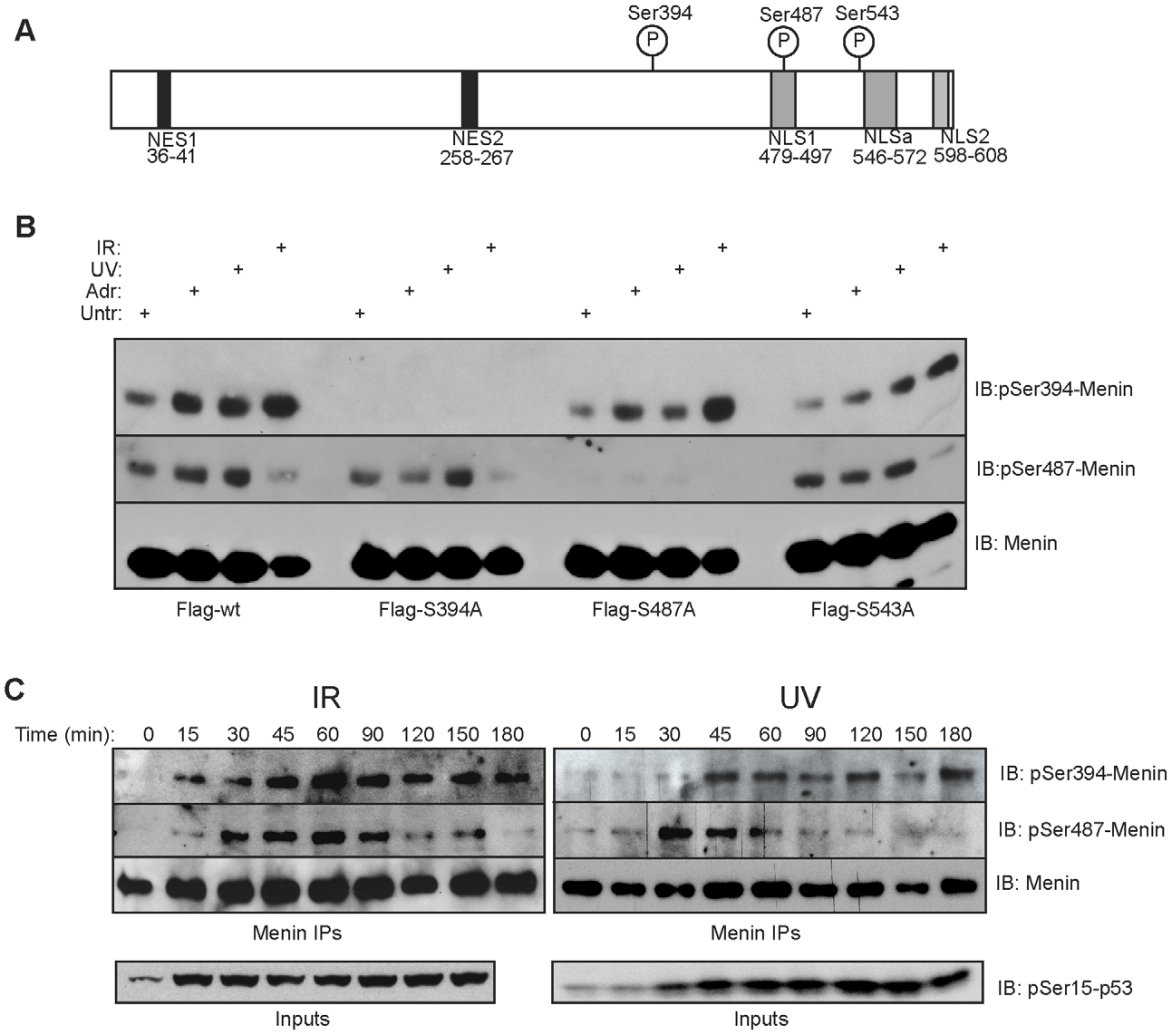
Menin is phosphorylated following DNA damage. (A) Schematic diagram of menin showing locations of detected phosphorylation sites relative to nuclear export (NES) and nuclear localization sequences (NLS). (B) Flag IPs from 293T cells transfected with Flag-wildtype menin, or Flag-phospho-deficient mutants following treatment with 0.05 uM Adr, 25J/m^2^ UV or 1000Rads of γ-IR. IPs were resolved and immunoblotted with phospho-Ser394, phospho-Ser487 or total menin. (C) Menin IPs were performed over the course of 3 hours from 293T cells treated with 1000 Rads of γ-IR (left panel) or 25J/m^2^ UV (right panel) and immunoblotted with phospho-Ser394, phospho-Ser487 or total menin. Whole 293T cell lysates were probed for phospho-Ser15-p53 over the same time course (lower panel).

In order to investigate the significance of these phosphorylation sites, phospho-specific antibodies were generated against phospho-peptides corresponding to each of these sites. To examine the function of each phosphorylation site, and to confirm the specificity of the phospho-specific antibodies, we generated Flag-tagged menin mutants with non-phosphorylatable Serine to Alanine substitutions at each site. We were unable to detect a signal with the phospho-Ser394 and phospho-Ser487 antibodies in Flag immunoprecipitations from cells expressing the Ser394Ala and Ser487Ala mutants, respectively, demonstrating antibody specificity (Figure 4B and S3A). Cells transiently expressing these phospho-deficient mutants were treated with Adr, UV or γ-IR and differences in phosphorylation at the remaining unsubstituted phosphorylation sites were evaluated (Figure 4B). The Flag-tagged wild type, Ser487Ala and Ser543Ala menin expressing cells all showed a strong induction of Ser394 phosphorylation after DNA damage, with γ-IR causing the strongest induction despite lower levels of immunoprecipitated menin as evaluated by total menin immunoblots. A low level of Ser394 phosphorylation was found in untreated cells. Phosphorylation of Ser487 was comparable between Flag-tagged wild type, Ser394Ala and Ser543Ala menin expressing cells.

Investigation into the phosphorylation status of endogenous menin in 293T cells exposed to different forms of DNA damage confirmed the induction of Ser394 phosphorylation while the levels of Ser487 and Ser543 phosphorylation appeared to be constant across treatment conditions (Figure S3B). We next examined the phosphorylation kinetics of these sites following treatment with either γ-IR or UV. Ser394 phosphorylation was detectable after only 15 minutes and remained elevated for at least 3 hours following γ-IR (Figure 4C). UV treatment induced Ser394 phosphorylation first became detectable after 45 minutes and remained elevated for at least 3 hours. Ser487 phosphorylation was found to be more dynamic, with γ-IR triggering a sharp increase followed by a gradual decline over time. UV treatment however, showed a peak of Ser487 phosphorylation by 30 minutes, followed by a rapid decrease in detectable levels. For comparison, DNA damage induced phosphorylation of p53 at Ser15 is shown after both treatments (Figure 4C, lower panel). These data demonstrate that menin is phosphorylated and dephosphorylated at different rates following γ-IR and UV, which may be important in mediating protein-protein interactions.

To understand whether or not phosphorylation of Ser394 was dependent upon Ser487 and vice versa, a time course study was performed using 293T cells transiently transfected with Flag-tagged wild type menin, Ser394Ala or Ser487Ala point mutants and subjected to either γ-R or UV. Treatment of cells expressing Flag-Ser487Ala with either γ-IR or UV led to an increase in detectable phosphorylation of Ser394, however the duration of the signal was attenuated after γ-IR while found to be more intense after UV treatment as compared to Flag-tagged wild type menin (Figure S4A). In Flag-Ser394Ala expressing cells, Ser487 phosphorylation levels were elevated for a more prolonged duration following both γ-IR and UV treatment as compared to wild type (Figure S4B). These results suggest that the phosphorylation of Ser394 may also be important in the dephosphorylation of Ser487 and that a complex interplay may exist between the phosphorylation of Ser394 and Ser487.

These results demonstrate that menin undergoes at least three post-translational modifications, with Ser394 phosphorylation being DNA-damage dependent. Both Ser543 and Ser487 phosphorylation were present under normal cell culture conditions, however an increase in Ser487 phosphorylation does occur following DNA damage.

### Menin Ser394 phosphorylation is dependent upon ATM and ATR

**Figure 5:**
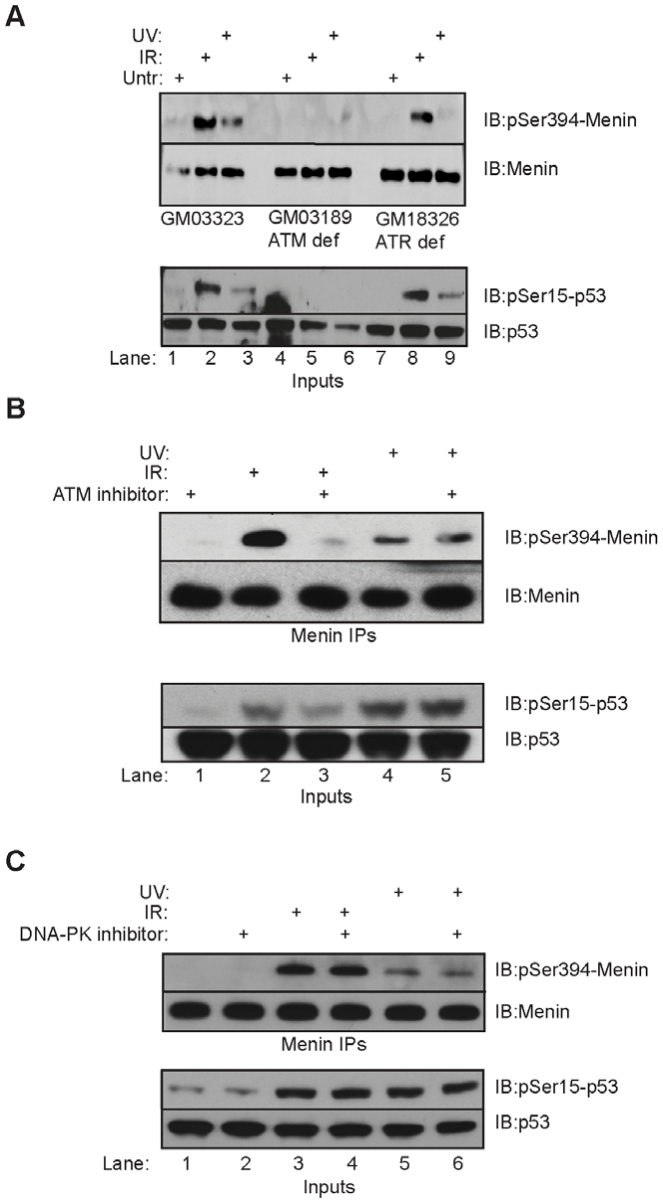
Menin phosphorylation is dependent upon ATM and ATR. (A) Menin IPs from control GM03323, ATM deficient GM03189 and ATR deficient GM18326 lymphoblastoid cells were performed 90 minutes after exposure to 1000 Rads γ-IR or 30 minutes after 25J/m^2^ UV. IPs were resolved and immunoblotted with phospho-Ser394 or total menin antibodies. Whole cell extracts from each cell type were immunoblotted with phospho-Ser15-p53 and total p53 (lower panel). (B) 293T cells were pretreated with 10 uM KU55933 for 2 hours prior to treatment with 1000 Rads γ-IR or 25J/m^2^ UV. Menin IPs were performed, resolved and immunoblotted for phospho-Ser394 or total menin. The same whole cell extracts were immunoblotted with phospho-Ser15-p53 and total p53 (lower panel). (C) 293T cells were pretreated with 10 uM DNA-PK inhibitor II for 2 hours prior to treatment with 1000 Rads γ-IR or 25J/m^2^ UV. Menin IPs were performed, resolved and immunoblotted for phospho-Ser394 or total menin. The same whole cell extracts were immunoblotted with phospho-Ser15-p53 and total p53 (lower panel).

The DNA damage transducing kinases ATM, ATM- and Rad3-related (ATR) and DNA-Protein Kinase (DNA-PK) are activated after damage and phosphorylate downstream targets containing SQ/TQ recognition motifs. To determine whether menin Ser394 phosphorylation was dependent upon ATM and/or ATR following DNA damage we utilized cells deficient in these enzymes. Following γ-IR treatment, there was no detectable phospho-Ser394 in ATM-deficient GM03189 cells while a strong signal was detected in the ATR-deficient GM18326 cells and control GM03323 cells (Figure 5A, upper panel, lanes 2, 5 and 8). In contrast, UV treatment resulted in a strong induction of Ser394 phosphorylation only in control cells while very weak signals were seen in both ATM-and ATR-deficient cells. Phosphorylation of p53 at Ser15 was also abrogated in the ATM deficient cell line after γ-IR and UV, indicating the importance of ATM signaling after both forms of damage (Figure 5A, lower panel).

To examine further whether Ser394 phosphorylation was dependent upon ATM, 293T cells were pretreated with the ATM inhibitor KU55933 prior to γ-IR or UV treatment. A dramatic decrease in detectable Ser394 phosphorylation was seen in cells treated with KU55933 after γ-IR when compared to γ-IR alone (Figure 5B, upper panel, lanes 2 and 3). No detectable change was observed after UV treatment in the presence or absence of KU55933. Cells pretreated with KU55933 showed a reduction in p53-Ser15 phosphorylation after γ-IR treatment but not UV (Figure 5B, lower panel). Pretreatment with a DNA-PK inhibitor did not influence menin-Ser394 phosphorylation after either γ-IR or UV exposure (Figure 5C, upper panel, lanes 4 and 6). These results indicate that γ-IR induced menin Ser394 phosphorylation is dependent upon ATM, and that UV-induced menin phosphorylation is dependent upon ATR.

To determine if menin half-life is extended following DNA damage, we next treated cells with γ-IR and added the protein synthesis inhibitor cycloheximide (CHX). In cells treated with CHX alone, menin had a half-life of approximately 45 minutes, while cells treated with γ-IR and CHX, menin half-life was extended for at least 2 hours (Figure S4C). These data differ from the half-life reported by Yaguchi et al [31]. The likely explanation is because the previous study analyzed either transfected menin and/or analysis of methionine-labeled protein rather than unlabeled whole cell lysate, and these differences in approach and cell culture conditions may cause variations in the measurement of protein. This suggests that DNA-damage may either stabilize menin protein levels or disrupt the pathways involved in menin degradation.

### MEN1-associated missense menin mutants show altered Ser394 and Ser487 phosphorylation

**Figure 6:**
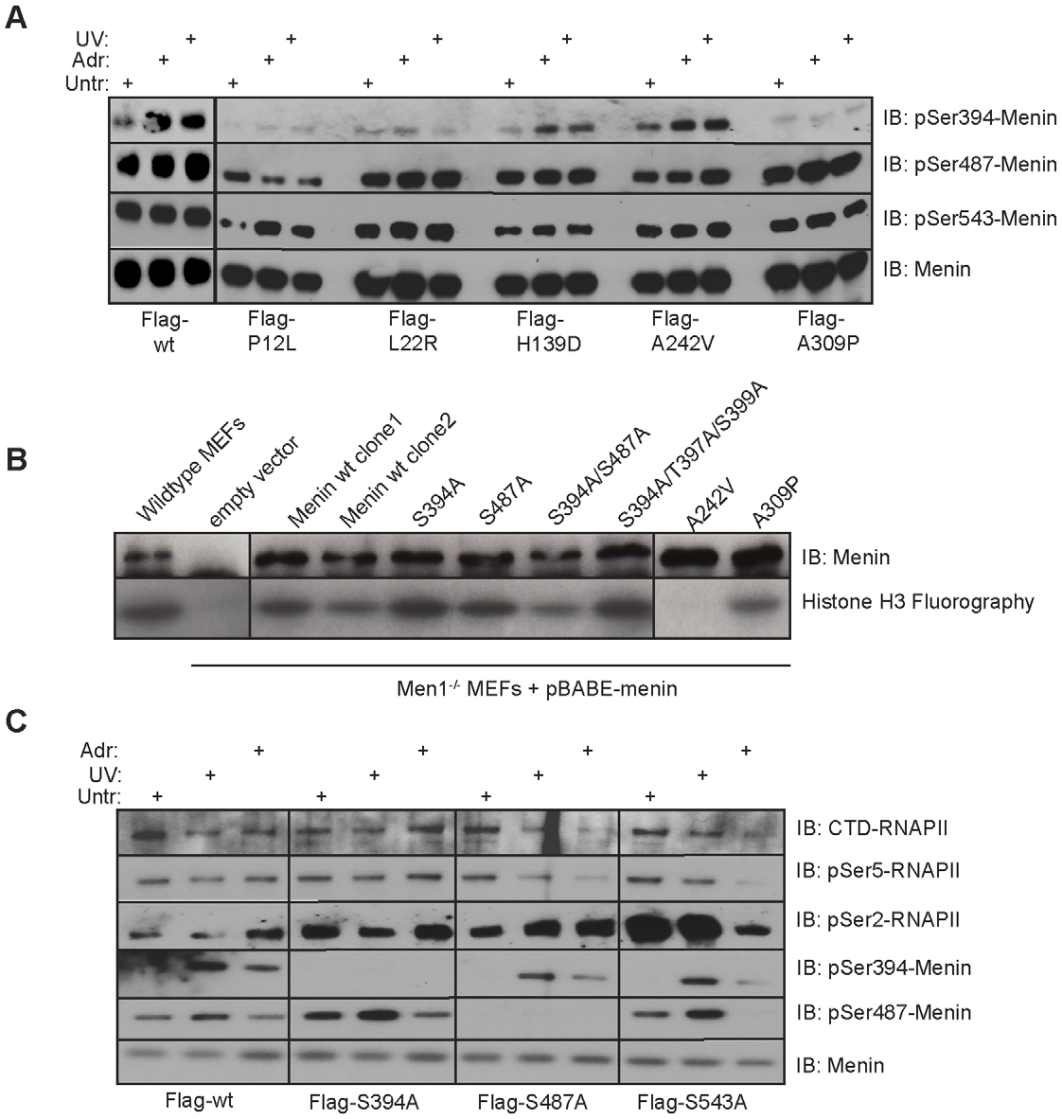
MEN1 missense point mutants display altered phosphorylation. (A) Flag IPs from293T cells transfected with Flag-wildtype menin, or Flag missense mutants following treatment with 0.05uM Adr for 18h, or 30 minutes after 25J/m^2^ UV. IPs were immunoblotted with phospho-Ser394, phospho-Ser487, phospho-Ser543 or total menin. (B) Menin IPs from Men1^−/−^ MEFs expressing wildtype or phospho-deficient point mutants were incubated with histone H3 and the methyl donor ^3^H-SAM to assay for histone methyltransferase activity. Reactions were resolved on 15% SDS-PAGE, amplified, dried and fluorographed. (C) Flag IPs from 293T cells transfected with phospho-deficient point mutants 30 minutes following treatment with 25 J/m^2^ UV or 18 h following 0.05 uM Adr, were resolved and immunoblotted with phospho-menin antibodies or phospho-RNAPII antibodies.

A major question in the menin field is the link between missense point mutations and their loss of various menin functions— in particular, some but not all of these mutants lack the ability to interact with KMT2A/KMT2D [12]. To determine whether or not MEN1-associated missense point mutants are phosphorylated, we screened a panel of mutants for the ability to undergo Ser487, Ser543 and Ser394 phosphorylation. Flag-tagged point mutants were immunoprecipitated from transiently transfected 293T cells following treatment with Adr or UV. As shown in Figure 6A, the mutants P12L, L22R, and A309P fail to achieve the level of Ser394 phosphorylation that is observed in the wild type menin and the H139D and A242Vmutants. In addition, the P12L mutant also displayed altered Ser487 phosphorylation following UV treatment (Figure 6A).

Interestingly, the MEN1-associated mutants found to be phosphorylated at Ser394, H139D and A242V, are also the tumor mutants that have been shown to not have the ability to interact with histone methyltransferase activity (HMTase) [12]. To examine whether or not Ser394 and/or Ser487 phosphorylation impacts the ability of menin to interact with HMTase activity, Men1^−/−^ mouse embryonic fibroblasts (MEFs) stably expressing wild type menin, or phospho-deficient menin mutants were analyzed. Menin immunoprecipitations were tested for HMT activity through the transfer of a radiolabelled methyl group to histone H3 (Figure 6B). We found that the Ser394Ala, Ser487Ala, Ser394/Ser487Ala and Ser394/Thr397/Ser399Ala menin mutants were still able to immunoprecipitate HMT activity. The MEN1-associated missense mutant A309P maintained the interaction with HMT activity while the A242V mutant did not, consistent with previous reports [12]. These results strongly suggest that Ser394 and Ser487 phosphorylation do not mediate or interfere with the interaction between menin and KMT2A/KMT2D. We also detected a number of peptides corresponding to KMT2A/KMT2D in menin immunoprecipitations following treatment with γ-IR and UV (Figure S5). The Ser543Ala mutant has been previously reported to not impact the interaction of menin with KMT2A and KMT2B as well [26].

A number of different approaches were undertaken to gain insight into the functional significance of these menin phosphorylation sites including BrdU incorporation and proliferation in Men1^−/−^ MEF cells expressing the Ser-to-Ala menin mutants; however, the mutants did not display significant alterations compared to wild type menin in these assays. We also examined changes in DNA damage-induced *Cdkn1A* expression and cell cycle effects in these MEF cells and were unable to detect any differences between menin null MEFs and those expressing wildtype or Ser-to-Ala mutations (data not shown).

We next looked to see if Ser394, Ser487 and Ser543 played a role in the interaction with RNAPII. Flag-tagged-wild type, -Ser394Ala, -Ser487Ala, or -Ser543Ala forms of menin were immunoprecipitated from 293T cells following treatment with either Adr or UV and the resulting complexes were immunoblotted for phosphorylated RNAPII. Under the conditions tested, phosphorylation of Ser-394 or Ser-487 did not correlate with either increased or decreased ability of menin to interact with RNAPII. The Flag-Ser543Ala menin mutant had a higher affinity for pSer2-RNAPII while the Flag-Ser394Ala and Ser487Ala mutants exhibited a modest increase in affinity for pSer2-RNAPII when compared to wild type menin in the absence of DNA damage (Figure 6C).

### Menin does not colocalize with γH2Ax foci

**Figure 7:**
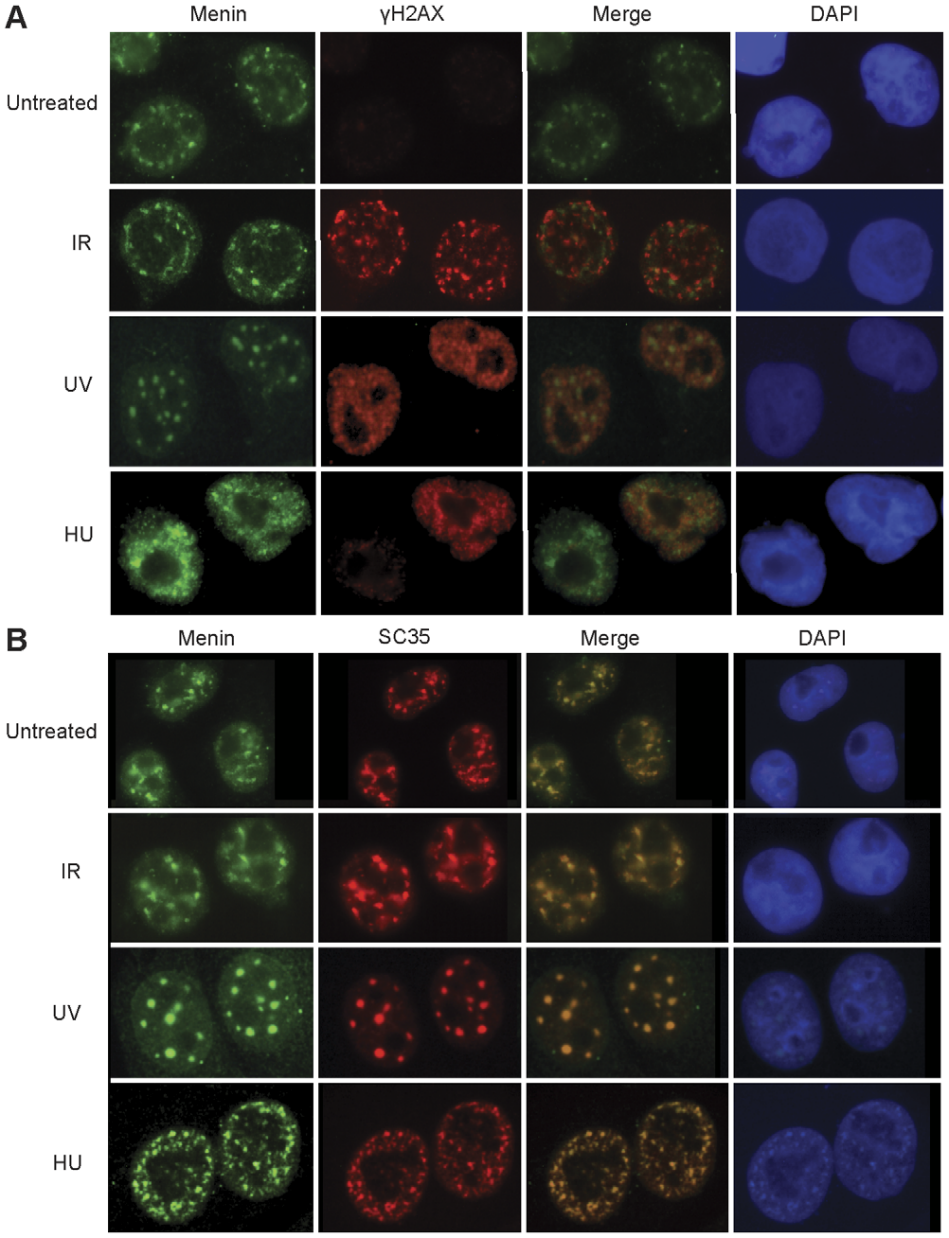
Menin does not colocalize with γ-H2AX foci. Immunofluorescence was performed in HeLa cells following treatment with 1000 Rads γ-IR (6 hr), 25 J/m^2^ UV (2hr) or 2 mM HU (18hr) and colocalization of menin with γ-H2AX (A) and SC35 (B) was assessed.

Several reports have suggested that menin may be involved in DNA repair [21], [24], [25]. To investigate a potential role for menin in the DNA repair process we performed immunofluorescence on cells treated with γ-IR, UV and HU and looked for menin relocalization to sites of DNA damage as visualized using phospho-Ser139 histone H2Ax (γH2Ax). We were unable to observe menin colocalization at γH2Ax foci in HeLa cells (Figure 7A) or in a number of other cell lines tested with additional menin antibodies (data not shown). An increase in menin staining at the nuclear matrix is observed in the γ-IR and UV treated cells consistent with previous reports [21], [32]. We attempted to visualize menin at DNA-damage foci using phospho-menin antibodies but the antibody specificity was not sufficient to distinguish between menin and other phosphorylated SQ motifs. Upon further investigation it was found that menin colocalized under all treatment conditions with SC35, a protein found within nuclear speckle domains in the nucleus (Figure 7B) [33].

## Discussion

Menin has long been implicated in the DNA damage response, either through direct interactions with DNA repair factors like FANCD2 and RPA or in the activation of genes involved in cell-cycle arrest or apoptosis [21], [22], [23], [24], [25]. Our results show that menin functions in the transcriptional response to DNA damage. We cannot conclusively determine whether or not menin plays an essential role in DNA repair given that functional experiments such as proliferation experiments in response to DNA damage showed no effect, and menin did not colocalize with γ-H2Ax foci, however it is possible that the conditions and cell lines tested in the study were not sensitive to menin perturbation. ChIP experiments have found that menin is associated with the 5′ regions of many DNA damage responsive genes in the absence of damage and this is suggestive of a mechanism where these important transcription components are “pre-loaded” in order to allow for immediate activation upon DNA damage [34]. Interestingly, we were able to see an interaction between menin and both the initiating (pSer5) and elongating (pSer2) forms of RNAPII and these data along with our ability to detect menin within the 3′ regions of these genes suggests that menin may associate with an elongation complex. Additional support for a role in elongation is provided by the colocalization of menin with nuclear speckle bodies as denoted by SC35. Nuclear speckles have been shown to contain both elongation and splicing factors, and menin could be interacting with any of the proteins involved in these nuclear bodies [35]. Furthermore, under conditions of elevated transcription, and increased menin association with promoter regions, we were unable to detect significant changes in histone methylation. This is suggestive of a function outside of histone methylation and consistent with the model proposed by Bres et al. whereby menin can activate transcription independently of KMT2A (MLL) [19]. It is also important to note that menin association with 5′ regions did not correlate with p53 binding suggesting that the two transcription regulatory factors function independently.

During the course of these studies we were able to confirm that menin undergoes a DNA damage dependent phosphorylation at Ser394. When Ser394 was mutated to an alanine, phosphorylation was also detected at either Thr397 or Thr399 suggesting that any SQ/TQ cluster can be modified. We were also able to identify an additional phosphorylation site at Ser487 and to confirm the phosphorylation of Ser543 [26]. Phosphorylation of Ser487 was dynamically regulated after DNA damage suggesting a tightly regulated function for this modification. Ser543 did not appear to undergo any noticeable change in phosphorylation level in the presence or absence of DNA damage and may be a constitutive phosphorylation site. The presence of Ser487 and Ser543 phosphorylation on menin purified from a mouse β cell line suggest that these modifications are likely to be important in menin function in the neuroendocrine lineages.

MEN1-associated missense menin point mutants can be classified based upon their ability to interact with KMT2A/D and it is believed that those mutants that retain the ability to interact with histone methyltransferase complexes have defects in other functions [14]. Surprisingly, we observed that most of the point mutants that had HMT activity failed to be phosphorylated at Ser394 following Adr and UV treatment. The presence or absence of phosphorylation at these sites does not appear to aid or interfere with the ability of menin to interact with KMT2A/D or RNAPII and is supportive of the concept that these tumor mutants are defective in other functions. It is believed that phosphorylation of these sites may be important for stabilizing or destabilizing protein complexes between menin and other currently unknown factors, however what those complexes are remains elusive. It is also possible that menin Ser394 phosphorylation occurs solely because the DNA damage transducing kinases ATM, ATR and DNA-PK are promiscuous and will phosphorylate any protein containing an SQ/TQ cluster whether they are functionally important or not. The overall significance of these menin phosphorylation sites have remained elusive. Complete elucidation of this question is likely dependent upon a cellular system that faithfully recapitulates the DNA damage response and contains matched cells with or without menin.

## Materials and Methods

### Cell Culture and treatments

293T, HeLa, U2OS (ATCC), βTC3 [36], and Men1^−/−^ MEF cells were cultured in DMEM supplemented with 10% fetal bovine serum (FBS), 1% Pen/Strep and 1% L-Glu. Lymphoblastoid (Coriell Cell Repository) cells were cultured in RPMI supplemented with 20% FBS, 1% Pen/Strep and 1% L-Glu. Gamma Irradiation treatment was performed using Cs^137^ source. UV treatment was performed using a Stratalinker 2400 (Stratagene). Adriamycin (Fisher) was used at 0.05 uM for 18 hours and hydroxyurea (Sigma) was used at 2 mM for 18 hours. KU55933 and DNA-PK Inhibitor II (EMD Biosciences) were used at 10 uM and added 2 hours prior to treatment with 1000 Rads γ-IR or 25 J/m^2^ UV. Cycloheximide (Sigma Aldrich) was used at 20 ug/ml and timepoints were harvested in lysis buffer.

### Vectors and transient transfections

pCDNA-Flag wildtype menin and MEN1 missense point mutants have been described [12]. pCDNA-Flag wildtype menin was used as a template to generate Ser-to-Ala mutants using Stratagene Site Directed mutagenesis. All mutations were confirmed by sequencing. 293T or HeLa cells were transfected using Fugene6 (Roche) using manufacturers protocol. Retroviral infections of Men1^−/−^ MEFs were done as previously reported [12].

### Antibodies

Phospho-menin antibodies were generated by Bethyl laboratories and affinity purified. Total menin (A300-105A) antibody was also from Bethyl Laboratories. Flag M2 (F3165), Vinculin (V4505) and SC35 (S4045) were from Sigma. Phospho-Ser15-p53 (9284), and phospho-Ser139-H2Ax (2577) were from Cell Signaling. Total RNAPII-CTD (MMS-126R), pSer5-CTD (MMS-134R) and pSer2-CTD (MMS-129R) were from Covance. p53 clones DO1 (SC-126X) and FL (SC-55476) were from Santa Cruz Biotechnology. Histone H3 trimethyl K4 (ab8580) was from Abcam.

### Whole cell extracts and immunoprecipitations

Extracts were prepared in IP lysis buffer as described in [12] containing Protease and Phosphatase Inhibitors (EMD Biosciences) and lysates were sonicated 3 times at setting 1 for 5s pulses with a Branson Sonifier 450.Samples were centrifuged for 20 minutes at maximum speed and the supernatants moved to new tubes. Protein concentrations were measured and equal levels were used for each immunoprecipitation. Antibody and Protein G beads (Amersham) were added to each supernatant and rocked with constant motion for 3 hours and then washed 4 times in 1 ml of IP lysis buffer. The resulting immunocomplexes were resuspended in 50 ul of 1X SDS loading buffer, boiled for 5 minutes and run on 4-20% Tris-Glycine gels (Invitrogen). The gels were transferred to nitrocellulose and probed with indicated antibodies.

### Immunoprecipitation histone methyltransferase assay

These studies were performed as described [12].

### Immunofluorescence

These studies were performed as described [37].

### Real time quantitative PCR

RNA was purified using RNeasy Columns (Qiagen) with DNase digestion. Superscript III RT (Invitrogen) and random hexamers were used to make cDNA. The Applied Biosystems 7500 real time PCR machine was used with Applied Biosystems Power Sybr master mix using an anneal/extend temperature of 60°. Relative expression was calculated using ΔΔCt. Statistical significance was calculated by two-tailed T-test comparing DNA damage with untreated conditions. Primers used for qRT-PCR are listed in Table S1.

### Chromatin Immunoprecipitation

ChIP was performed and analyzed as described [38], with the following modifications, chromatin was sheared using a Branson Sonifier 450 at power setting 4, 30% duty cycle for 8×1 min pulses to generate 200–600 base pair fragments, crosslinking was reversed overnight at 65°C in elution buffer, supernatant was diluted in an equal volume TE buffer and digested with Proteinase K for 2 hr at 37°C. The DNA was purified using PCR Cleanup Columns (Qiagen) following manufacturers protocol and diluted 4 fold in TE. Quantitative PCR was performed in triplicate. A 1:1 mixture of p53 DO1 and FL antibodies were used for p53 IPs. Primers for the *CDKN1A* and *MDM2* [39], *GADD45A* [34]and also are listed in Table S1. The Applied Biosystems 7500 real time PCR machine was used with Applied Biosystems Power Sybr master mix using an anneal/extend temperature of 60° All PCR reactions were run in triplicate and the average Ct value was to calculate the percent input recovered using the formula  = 2∧(average input Ct-average antibody Ct). Statistical significance was calculated by a two-tailed T-test comparing the percentage of input DNA recovered in each immunoprecipitation with the no antibody control. All PCR primers amplified a single product of the appropriate size.

## Supporting Information

Figure S1 **Trimethylation of histone H3 is not significantly increased after IR.** ChIPs were performed in U2OS cells 6 hours following treatment with 1000 Rads of γ-IR and immunoprecipitated with an antibody recognizing histone H3K4me3. Precipitated chromatin was used for quantitative real-time PCR using primers that amplify 5′ regions of the indicated genes. Results are the average of at least 3 independent experiments and represent the amount of DNA immunoprecipitated with each antibody relative to the amount of input DNA. Error bars indicate standard error of the mean.
(TIF)

Figure S2 **Menin mass spectrometry data.** (A) Endogenous menin was immunoprecipitated from untreated 293T cells or 18 hours after addition of 2 mM Hydroxyurea, 6 hours after 1000 Rads of γ-IR, or 2 hours after exposure to 25 J/m^2^ UV. The identified phospho-peptides are listed by treatment conditions with the predicted Serine site in bold. Coverage indicates peptides corresponding to total protein analyzed by mass spec. (B) Endogenous menin was immunoprecipitated from the mouse β cell line βTC3. The identified phospho-peptides are listed. (C) Alignment of identified phosphorylation sites with menin sequences from other metazoans using NCBI COBALT alignment software.
(TIF)

Figure S3 **Menin phosphorylation after DNA damage.** (A) Flag IPs from 293T cells transfected with Flag-wildtype menin, or Flag-phospho-deficient mutants harvested 2 hours after treatment with 1000 Rads of γ-IR. IPs were resolved and immunoblotted with phospho-Ser394, phospho-Ser543 or total menin. (B) Endogenous menin was immunoprecipitated from untreated 293T cells or 2 hours after treatment with 25 J/m^2^ UV, 18 hours after addition of 2 mM Hydroxyurea, 18 hours after addition of 0.05 uM Adr, or 2 hours after 1000 Rads of γ-IR. Immunoprecipitates were resolved and immunoblotted with phospho-specific antibodies. (B) Time course immunoprecipitations of Flag-menin wildtype or Flag-Ser487Ala mutant after 1000 Rads of γ-IR or 25 J/m^2^ UV treatment and immunoblotted with phospho-specific antibodies. (C) Time course immunoprecipitations of Flag-menin wildtype or Flag-Ser394Ala mutant after 1000 Rads of γ-IR or 25 J/m^2^ UV treatment and immunoblotted with phospho-specific antibodies. (D) 293T whole cell extracts from cells treated with 1000 Rads of γ-IR in the presence or absence of 20 ug/mL CHX and immunoblotted for menin and Vinculin as a loading control.
(TIF)

Figure S4 **Menin Ser-to-Ala mutant phosphorylation kinetics.** (A) Time course immunoprecipitations of Flag-menin wildtype or Flag-Ser487Ala mutant after 1000 Rads of γ-IR or 25 J/m^2^ UV treatment and immunoblotted with phospho-specific antibodies. (B) Time course immunoprecipitations of Flag-menin wildtype or Flag-Ser394Ala mutant after 1000 Rads of γ-IR or 25 J/m^2^ UV treatment and immunoblotted with phospho-specific antibodies. (C) 293T whole cell extracts from cells treated with 1000 Rads of γ-IR in the presence or absence of 20 ug/mL CHX and immunoblotted for menin and Vinculin as a loading control.
(TIF)

Figure S5 **Menin coimmunoprecipitation mass spectrometry data.** Endogenous menin was immunoprecipitated from untreated 293T cells or 6 hours after 1000 Rads of γ-IR, or 2 hours after exposure to 25 J/m^2^ UV. The resulting immunoprecipitates were resolved and prominent bands were excised for mass spectrometry. The identified peptides from KMT2A/KMT2D and subunits of RNA Polymerase II are shown.
(TIF)

Table S1 **Primers for qRT-PCR and ChIP used in this study.**
(DOC)
