# Supplementary figures and images for: The Menin Tumor Suppressor Protein Is Phosphorylated in Response to DNA Damage
Source: PLoS One. 2011 Jan 14;6(1):e16119. doi: 10.1371/journal.pone.0016119 (PMC3021530; doi:10.1371/journal.pone.0016119)

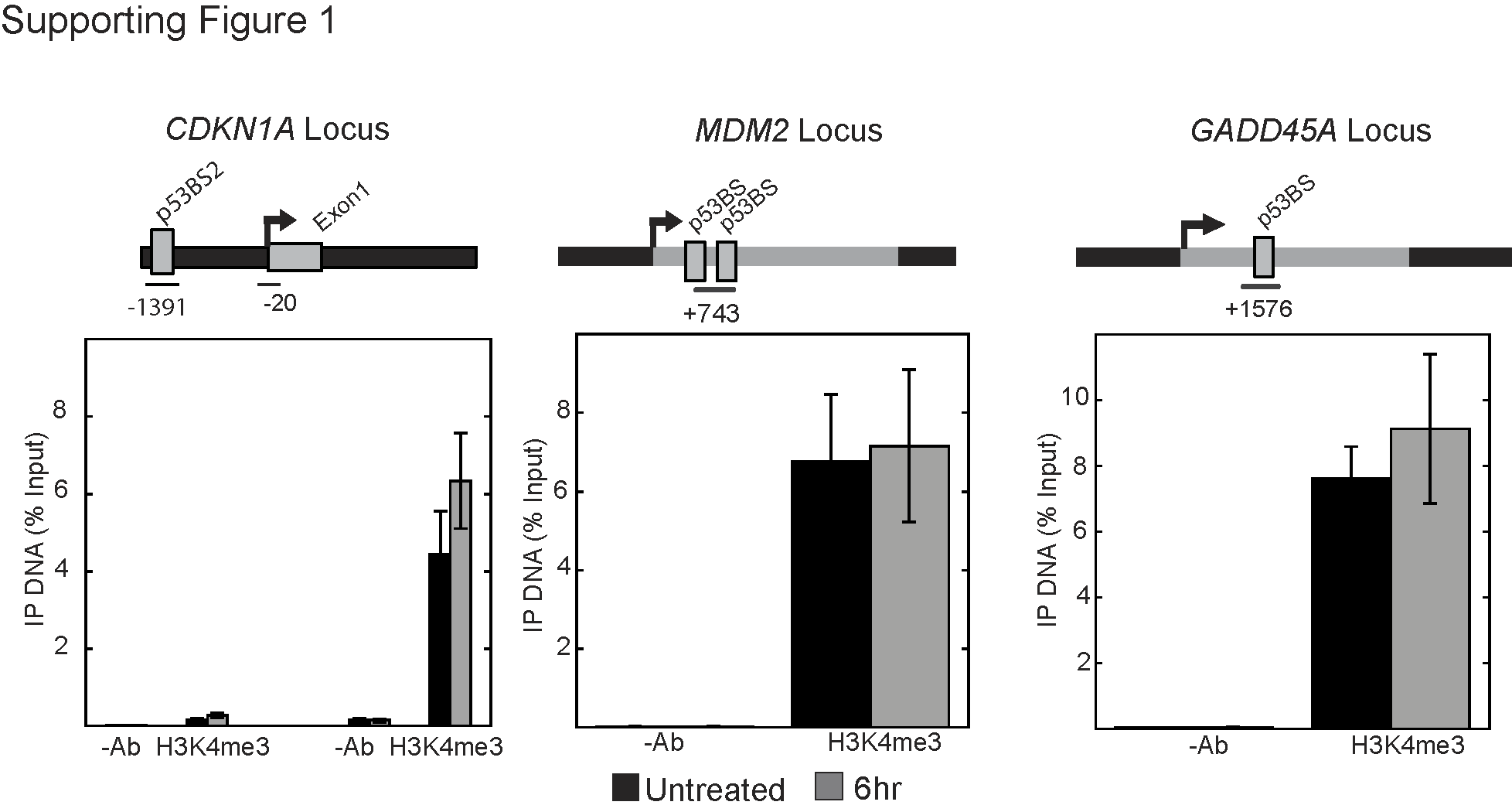

Supplement: Figure S1 — Trimethylation of histone H3 is not significantly increased after IR. ChIPs were performed in U2OS cells 6 hours following treatment with 1000 Rads of γ-IR and immunoprecipitated with an antibody recognizing histone H3K4me3. Precipitated chromatin was used for quantitative real-time PCR using primers that amplify 5′ regions of the indicated genes. Results are the average of at least 3 independent experiments and represent the amount of DNA immunoprecipitated with each antibody relative to the amount of input DNA. Error bars indicate standard error of the mean. (TIF) [file pone.0016119.s001.tif]

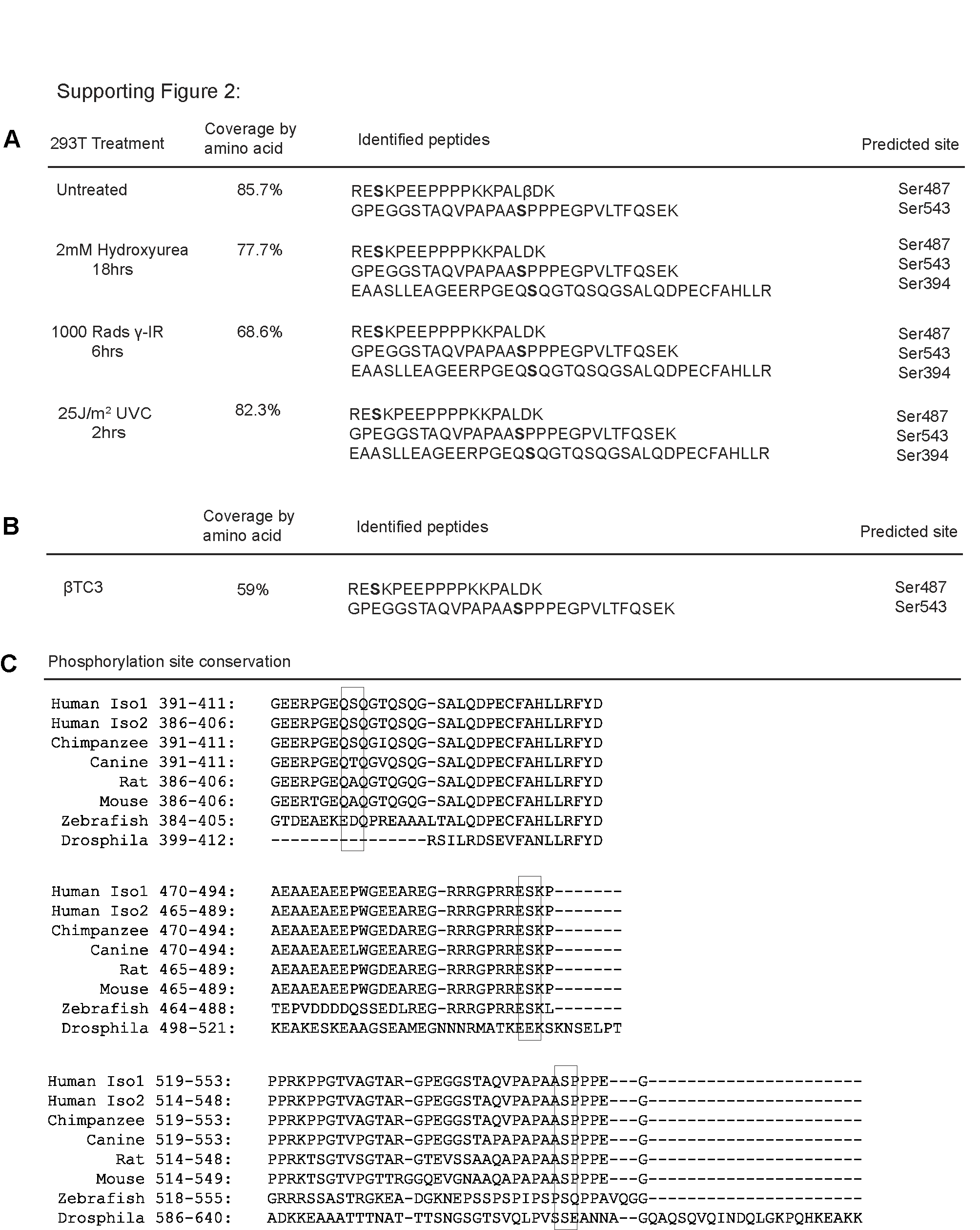

Supplement: Figure S2 — Menin mass spectrometry data. (A) Endogenous menin was immunoprecipitated from untreated 293T cells or 18 hours after addition of 2 mM Hydroxyurea, 6 hours after 1000 Rads of γ-IR, or 2 hours after exposure to 25 J/m2 UV. The identified phospho-peptides are listed by treatment conditions with the predicted Serine site in bold. Coverage indicates peptides corresponding to total protein analyzed by mass spec. (B) Endogenous menin was immunoprecipitated from the mouse β cell line βTC3. The identified phospho-peptides are listed. (C) Alignment of identified phosphorylation sites with menin sequences from other metazoans using NCBI COBALT alignment software. (TIF) [file pone.0016119.s002.tif]

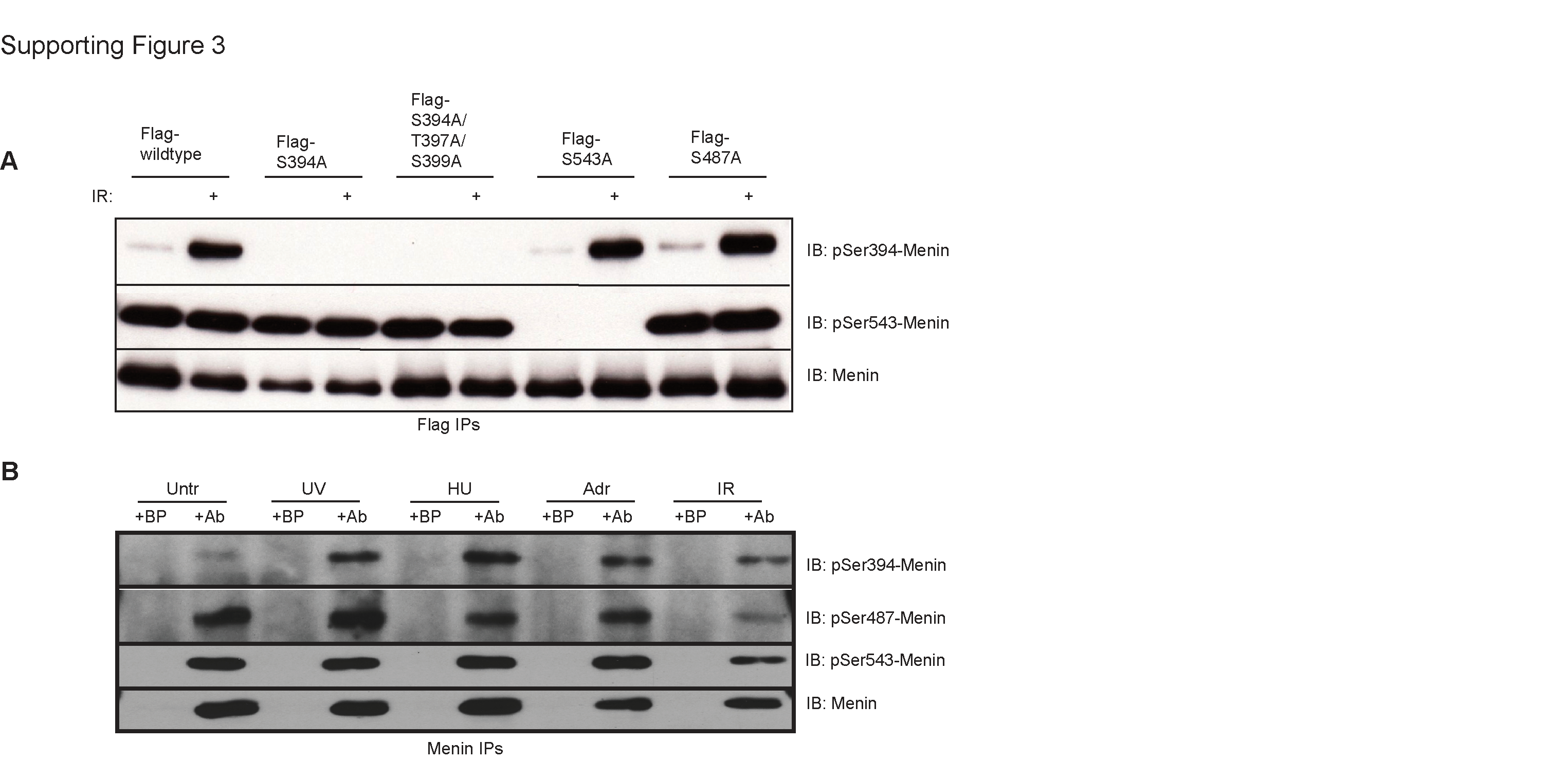

Supplement: Figure S3 — Menin phosphorylation after DNA damage. (A) Flag IPs from 293T cells transfected with Flag-wildtype menin, or Flag-phospho-deficient mutants harvested 2 hours after treatment with 1000 Rads of γ-IR. IPs were resolved and immunoblotted with phospho-Ser394, phospho-Ser543 or total menin. (B) Endogenous menin was immunoprecipitated from untreated 293T cells or 2 hours after treatment with 25 J/m2 UV, 18 hours after addition of 2 mM Hydroxyurea, 18 hours after addition of 0.05 uM Adr, or 2 hours after 1000 Rads of γ-IR. Immunoprecipitates were resolved and immunoblotted with phospho-specific antibodies. (B) Time course immunoprecipitations of Flag-menin wildtype or Flag-Ser487Ala mutant after 1000 Rads of γ-IR or 25 J/m2 UV treatment and immunoblotted with phospho-specific antibodies. (C) Time course immunoprecipitations of Flag-menin wildtype or Flag-Ser394Ala mutant after 1000 Rads of γ-IR or 25 J/m2 UV treatment and immunoblotted with phospho-specific antibodies. (D) 293T whole cell extracts from cells treated with 1000 Rads of γ-IR in the presence or absence of 20 ug/mL CHX and immunoblotted for menin and Vinculin as a loading control. (TIF) [file pone.0016119.s003.tif]

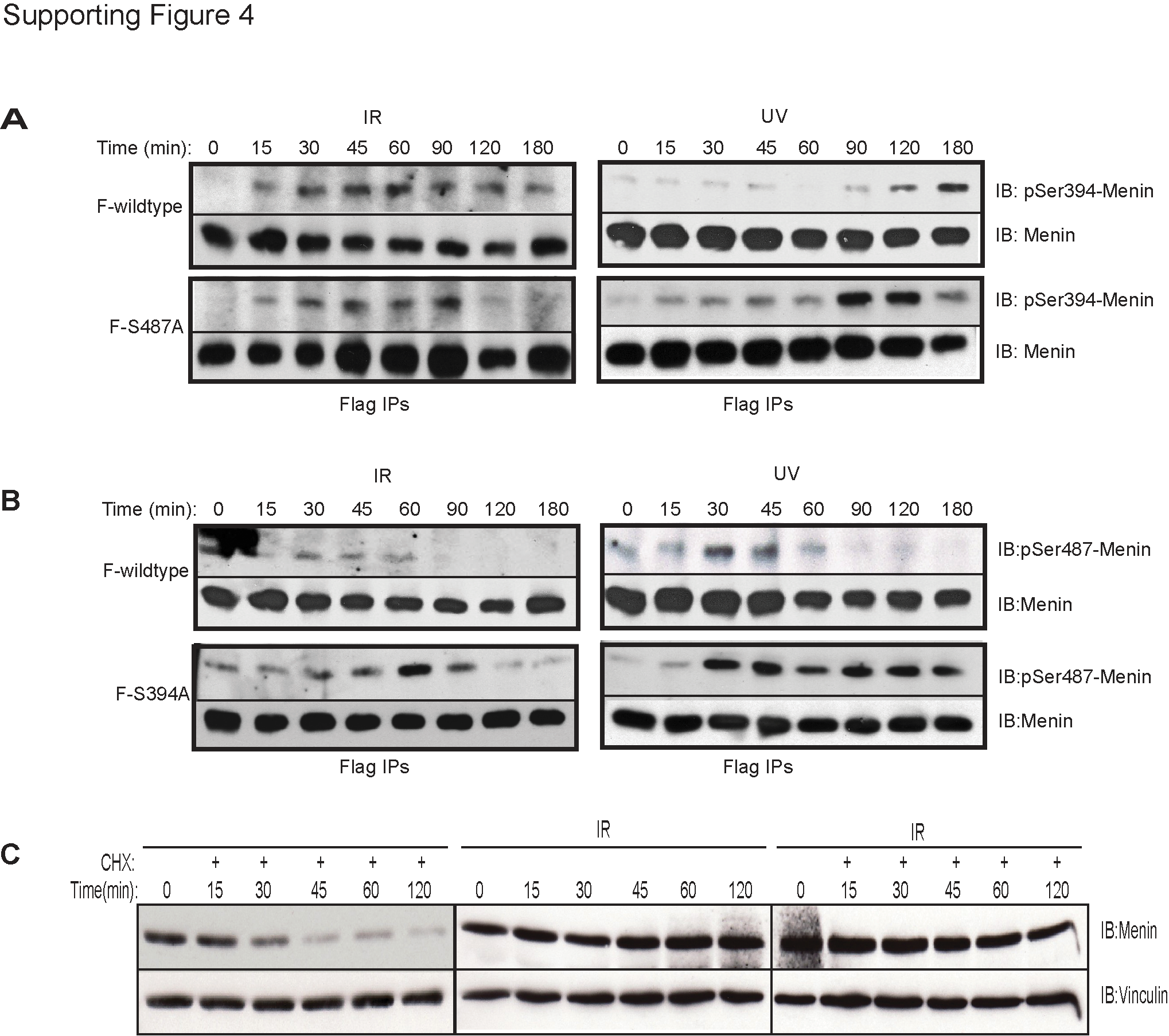

Supplement: Figure S4 — Menin Ser-to-Ala mutant phosphorylation kinetics. (A) Time course immunoprecipitations of Flag-menin wildtype or Flag-Ser487Ala mutant after 1000 Rads of γ-IR or 25 J/m2 UV treatment and immunoblotted with phospho-specific antibodies. (B) Time course immunoprecipitations of Flag-menin wildtype or Flag-Ser394Ala mutant after 1000 Rads of γ-IR or 25 J/m2 UV treatment and immunoblotted with phospho-specific antibodies. (C) 293T whole cell extracts from cells treated with 1000 Rads of γ-IR in the presence or absence of 20 ug/mL CHX and immunoblotted for menin and Vinculin as a loading control. (TIF) [file pone.0016119.s004.tif]

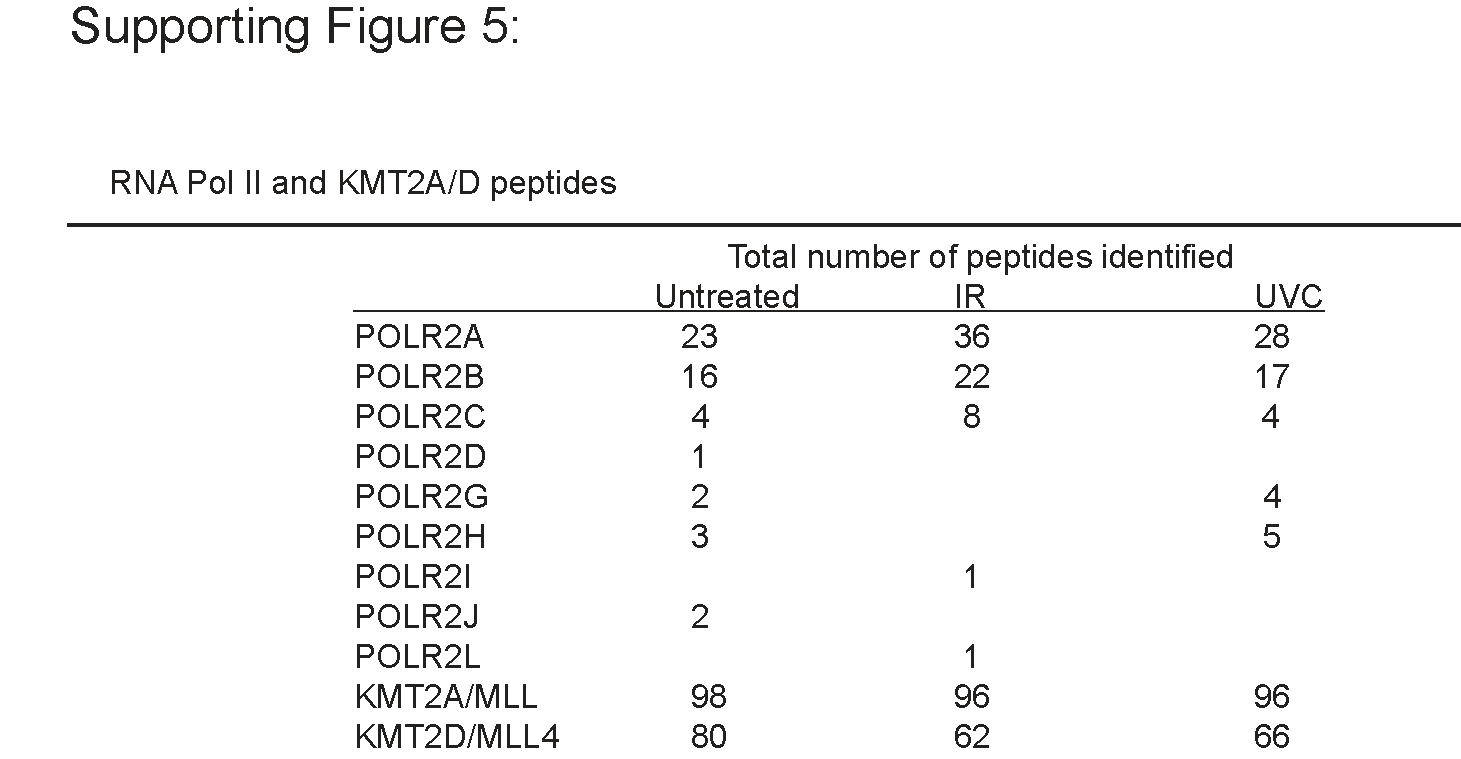

Supplement: Figure S5 — Menin coimmunoprecipitation mass spectrometry data. Endogenous menin was immunoprecipitated from untreated 293T cells or 6 hours after 1000 Rads of γ-IR, or 2 hours after exposure to 25 J/m2 UV. The resulting immunoprecipitates were resolved and prominent bands were excised for mass spectrometry. The identified peptides from KMT2A/KMT2D and subunits of RNA Polymerase II are shown. (TIF) [file pone.0016119.s005.tif]
