# Supplementary material for: The Menin Tumor Suppressor Protein Is Phosphorylated in Response to DNA Damage
Source: PLoS One. 2011 Jan 14;6(1):e16119. doi: 10.1371/journal.pone.0016119 (PMC3021530; doi:10.1371/journal.pone.0016119)
Supplement: Table S1 — Primers for qRT-PCR and ChIP used in this study. (DOC) [file pone.0016119.s006.doc]

| CACCGAGACACCACTGGAGG | human CDKN1A forward |  |
| --- | --- | --- |
| GAGAAGATCAGCCGGCGTTT | human CDKN1A reverse |  |
| TGCGAGAACGACATCAACAT | human GADD45A forward |  |
| TCCCGGCAAAAACAAATAAG | human GADD45A reverse |  |
| ATCTTGGCCAGTATATTATG | human MDM2 forward |  |
| GCTCTTTCACAGAGAAGC | human MDM2 reverse |  |

Table S1

RT-PCR primers

ChIP primers

| AGCAGGCTGTGGCTCTGATT | human CDKN1A -2283 forward |
| --- | --- |
| CAAAATAGCCACCAGCCTCTTCT | human CDKN1A -2283 reverse |
| CTGTCCTCCCCGAGGTCA | human CDKN1A -1391 forward |
| ACATCTCAGGCTGCTCAGAGTCT | human CDKN1A -1391 reverse |
| TATATCAGGGCCGCGCTG | human CDKN1A -20 forward |
| GGCTCCACAAGGAACTGACTTC | human CDKN1A -20 reverse |
| AGTCACTCAGCCCTGGAGTCAA | human CDKN1A +4000 forward |
| GGAGAGTGAGTTTGCCCATGA | human CDKN1A +4000 reverse |
| CCTCCCACAATGCTGAATATACAG | human CDKN1A +8500 forward |
| AGTCACTAAGAATCATTTATTGAGCAC | human CDKN1A +8500 forward |
| gattgtggatctgtggtaggtg | human GADD45 5’ forward |
| GAATTAGTCACGGGAGGCAGTG | human GADD45 5’ reverse |
| tccacattcatctcaatggaagg | human GADD45A 3' forward |
| gtgtAGGGAGTAACTGCTTGAg | human GADD45A 3' reverse |
| GGTTGACTCAGCTTTTCCTCTTG | human MDM2 5’ forward |
| GGAAAATGCATGGTTTAAATAGCC | human MDM2 5’ reverse |
| CTTTCTCGAGGAGGCAGGTTT | human MDM2 3’ forward |
| GCTCAACCCTAGGCGCTATTC | human MDM2 3’ reverse |
| ACACTAATCTATTACTGCGCTG | human HBG1 5’ forward |
| CCAGGATTTTTGACGGGAC | human HBG1 5’ reverse |
| AGAGCAGGTTTGTTGGCAGCA | human CDKN1B 5’ forward |
| ACAGAGGAGGAGATCCATTGG | human CDKN1B 5’ reverse |
| TGTCCATGGTGTGGATTTGCG | human BBC3 5’ forward |
| AGACACCGGGACAGTCGGACA | human BBC3 5’ reverse |
| ATGCCCATTTGTGCAACGA | human Fas 5' forward |
| CCTGCGCGCGGGTAG | human Fas 5' reverse |
| AGGAGGCGAGTGATAAGGATCC | human TP53I3 5’ forward |
| AACCTCTTGGCGGGCGGATTGG | human TP53I3 5’ reverse |
